# Comparison of the Chemical Composition and Antioxidant Properties of Propolis from Urban Apiaries

**DOI:** 10.3390/molecules28186744

**Published:** 2023-09-21

**Authors:** Katarzyna Pobiega, Anna M. Kot, Jarosław L. Przybył, Alicja Synowiec, Małgorzata Gniewosz

**Affiliations:** 1 Department of Food Biotechnology and Microbiology, Institute of Food Sciences, Warsaw University of Life Sciences WULS-SGGW, 159C Nowoursynowska Street, 02-776 Warsaw, Poland; anna_kot@sggw.edu.pl (A.M.K.); alicja_synowiec@sggw.edu.pl (A.S.); malgorzata_gniewosz@sggw.edu.pl (M.G.); 2 Department of Vegetable and Medicinal Plants, Institute of Horticultural Sciences, Warsaw University of Life Sciences WULS-SGGW, 159 Nowoursynowska Street, 02-776 Warsaw, Poland; jaroslaw_przybyl@sggw.edu.pl

**Keywords:** propolis, urban apiares, antioxidants, chemical composition

## Abstract

Bee products from urban apiaries are increasingly used. They are mainly used to promote local apiaries and cities in which they are located. The aim of the study was to compare the chemical composition and antioxidant activity of propolis from 6 Polish apiaries located in cities (Legionowo, Torun, Cracow, Warsaw, Katowice, Lodz). The chemical composition was analyzed using liquid chromatography (HPLC-DAD) and the analysis of antioxidant activity by scavenging free radicals (ABTS and DPPH) and FRAP. The obtained results showed the presence of 24 phenolic compounds in propolis extracts. The tested samples showed differentiation in terms of the content of individual chemical components, however, cinnamic acid and its derivatives were dominant. High antioxidant activity of the tested extracts was demonstrated (ABTS was in the range of 16.80–51.53 mg Te/mL, DPPH was in the range of 7.54–22.13 mg Te/mL, while FRAP reduction was in the range of 10.93–29.55 mg Te/mL). The obtained results compared with literature data on propolis from agricultural areas allow to conclude that propolis samples from both Poland types of areas are similar and can be classified as poplar propolis.

## 1. Introduction

Propolis is a natural mixture of resinous substances that has a waxy appearance and is collected in Europe by honeybees (*Apis mellifera*) from the leaves, buds and exudates of various plants, partially digested by β-glycosidase from the bees’ saliva and then mixed with beeswax [1]. The chemical composition of propolis is very complex, and the basic biologically active compounds are flavonoids, terpenes, aromatic acids and their esters. However, its composition is highly variable and depends on various factors such as geographical origin, types of plant sources, harvest time, season and climatic characteristics of the site [2,3].

Propolis has gained a worldwide reputation as a natural product that has gained widespread acceptance in many countries over the past few decades as a dietary supplement to support health and prevent disease. The health-promoting properties of propolis result from its chemical composition, which determines its versatile pharmacological action—including antibacterial, antifungal and antiviral properties, antioxidant, antiseptic, antimutagenic, hepatoprotective, anticancer and antidiabetic properties. In addition, it is also anti-inflammatory, cytostatic and immunostimulating, has a positive effect on the healing of wounds and burns. The richness of bioactive ingredients determines its use in medicine and dentistry, as well as in the pharmaceutical, cosmetic and food industries [4,5].

Due to the difficult situation associated with a significant decrease in the number of bees in areas outside cities, bee farms began to be introduced to large metropolises. The new trend started in 2007 and covered New York, London and Berlin [6,7]. Cities are perceived as a good shelter and a potential place for the development of bees, because in urban agglomerations they are much less exposed to harmful pesticides, which are used to a minimum extent in urban greenery compared to agricultural areas. In addition, cities often have a large variety of vegetation throughout the year, which has a positive effect on pollinating insects and, in the case of bees, allows them to produce numerous valuable products such as honey or propolis [8].

Urban beekeeping is developing dynamically, bringing many benefits. The factor influencing urban beekeeping is primarily location. It is a very important factor affecting successful harvests. Urban apiaries are most often located on the roofs of residential buildings, schools, organizations and enterprises, e.g., the roof of the Opera Garnier in Paris or the roofs of the National Opera and the Palace of Culture and Science in Warsaw. In London, the density of honey bee colonies was found to exceed 10 hives/km^2^, while the European average is 4.2 hives/km^2^ [9,10].

Honey bees are seen by many city dwellers as a symbol of prosperity and biodiversity of the ecosystem. Honey bee products can be biomonitors that offer effective environmental monitoring in cities. *A. mellifera* and their products provide geospatial resolution and time scale information. Biomonitors in this form are convenient due to the fact that *A. mellifera* is capable of multiplication and is found on almost all continents (except Antarctica), giving access to hives in all parts of the world [11]. Few studies of urban apiary products have shown that honeybees can collect other substances such as asphalt tar, paints or mineral oils when vegetation is scarce in the area, therefore it is important to provide suitable post-use bases for these insects [12].

Research on products from urban apiaries is mainly focused on honey, which is most often consumed. So far, there have been few studies of propolis from urban bee apiaries. In this study, the chemical compositions of extracts obtained from urban propolis were characterized, and their antioxidant properties were checked.

## 2. Results

### 2.1. Chemical Composition of Urban Propolis Extracts

A chromatographic analysis of seven ethanol propolis extracts from apiaries located on the roofs of buildings in cities was performed. The content of bioactive components of the tested extracts is presented in Table 1.

In urban propolis extracts, 25 chemical components were identified, including 24 phenolic acids and flavonoids and 1 alcohol (Table 1). UEP 1 and UEP 2 had the highest content of bioactive components in the dry matter of the extract, approx. 17 mg/100 mg d.m., UEP 7 had approx. 10.8 mg/100 mg d.m., while the lowest content of phenolic compounds was found in UEP 3 (at the level of 7 mg/100 mg d.m.) (Table 1). In urban propolis extracts, a large variation in the content of 3,4-dihydroxycinnamic acid (0.4–2 mg/100 mg d.m.), 4-hydroxy-3-methoxycinnamic acid (in the range from 0.1 to 5.3 mg/100 mg d.m.), ellagic acid dihydrate was observed (0.001–0.17 mg/100 mg d.m.) and 5,7-dihydroxyflavone (0.4–2.1 mg/100 mg d.m.). Catechin was present only in propolises from the cities of southern Poland (Cracow-UEP1, UEP3 and Katowice-UEP2) and was absent in propolises from other cities (Warsaw UEP 4, Legionowo UEP5, Lodz UEP6 and Torun UEP7). Ellagic acid dihydrate was present in higher amounts in UEP1 and UEP7 propolis extracts, while in UEP3-UEP6 it was present in minimal amounts.

**Figure 1:**
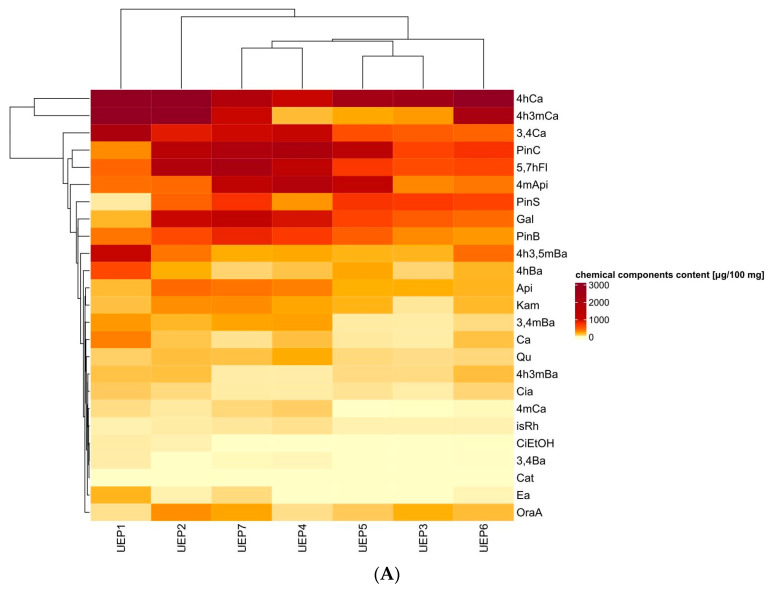

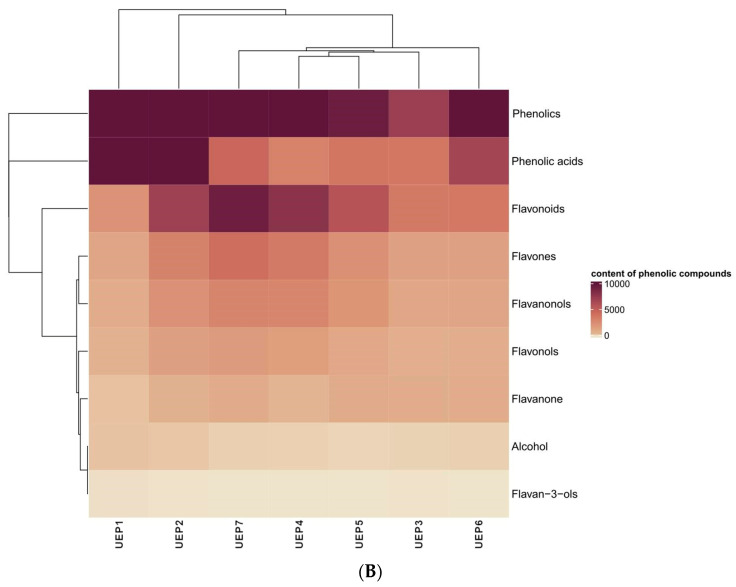
Heat map of (**A**) chemical composition of Polish urban propolis extract (light yellow to dark brown corresponding to a progressive increase in the content of component propolis extract) and (**B**) chemical group composition of Polish urban propolis extract.

The heat map (Figure 1A) identified the dominant components in the chemical profile of urban propolis and they were phenolic acids: 3,4-dihydroxycinnamic acid (4hCa), 4-hydroxy-3-methoxycinnamic acid (4h3mCa), 3,4-dihydroxycinnamic acid (3,4Ca). There were less flavonoids than phenolic acids, with the dominant flavonoids being 5,7-dihydroxyflavone (5,7hFl), methyl-4’-apigenin (4mApi) and pinocembrin (PinC). There were less total phenolics in UEP3 than in the other extracts (Figure 1B). More phenolic acids were found in UEP1 and UEP2 from two cities in southern Poland and UEP6 in central Poland (Lodz). More total flavonoids, including flavones and flavanols, were found in propolis from cities in central-northern Poland (Torun UPE7) and central Poland (Warsaw UPE4).

**Figure 2:**
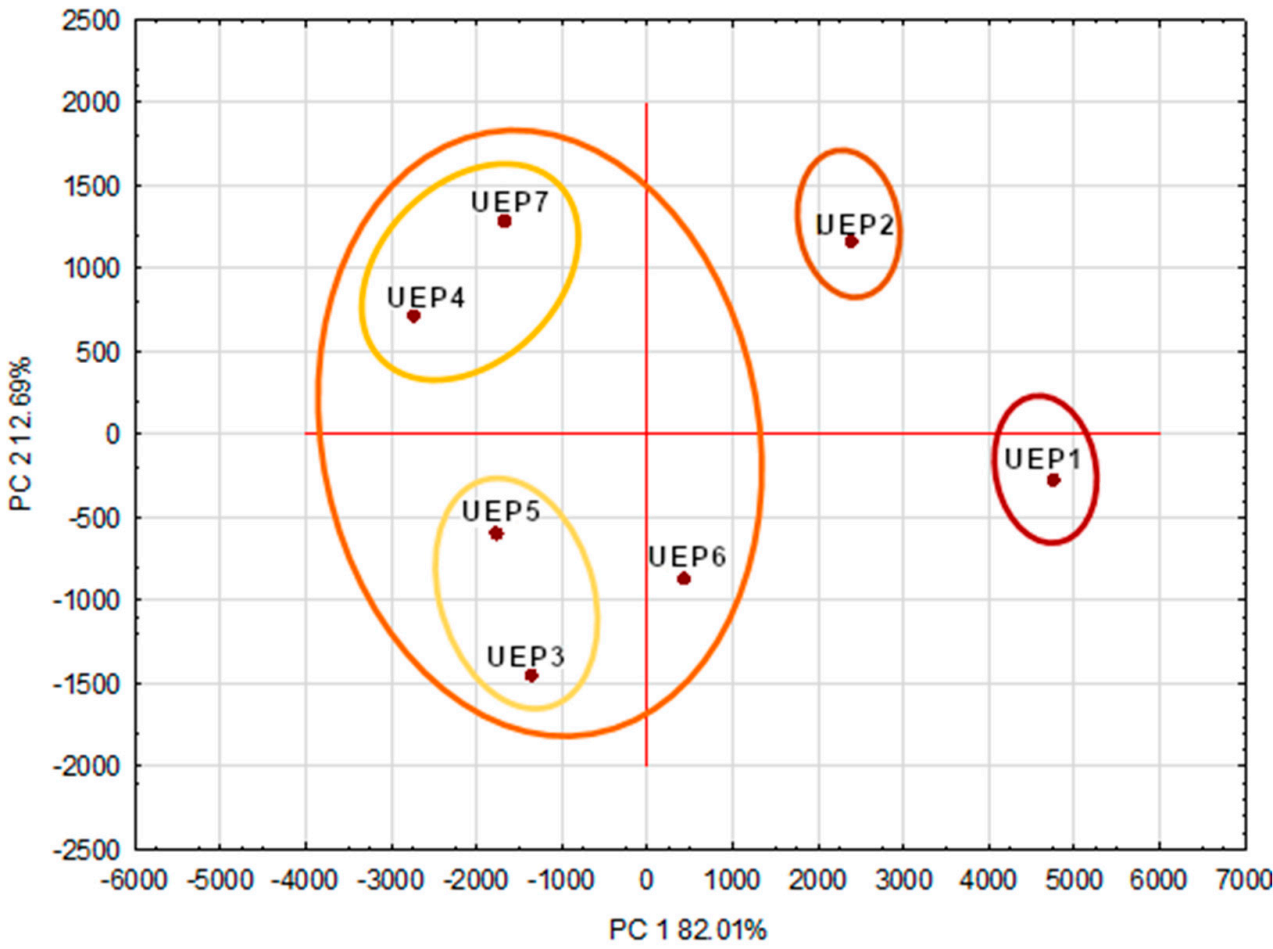
PCA map type propolis extract.

The PCA method (Figure 2) was used to compare urban propolis in terms of the content of biocomponents. Three different groups of urban propolis extracts appeared on the PCA map (PC1 82.01%, PC2 12.69%). Propolis from Cracow and Katowice, i.e., from southern Polish cities, differed from each other and from other propolis. UEP1 and UEP2 were characterized by a high content of phenolic acids, while they differed in the content of 5.7 dihydroxyflavone and pinocembrin, the content of which was significantly higher in UEP2 (Katowice). Propolis in the third group were characterized by a lower total content of phenolic acids (mainly 4h3mCa, 4hCa, 4h3,5mBa) and a higher content of flavonoids (5,7hFl, PinC, Gal, PinS) than UEP1 and UEP2.

Despite the different location of the cities from which they were obtained, the content of bioactive ingredients does not significantly differentiate these propolis. This may be due to the similar vegetation surrounding the buildings where the hives are located. The PCA map proves that the location of cities is a factor affecting the phenolic profile of propolis, in particular the content of some polyphenolic acids, and to a lesser extent the content of flavonoids and other polyphenolic components. The method of obtaining propolis from beehives had no effect on the profile of its bioactive components; propolis collected on gratings were classified to the same group as propolis collected from frames. Propolis obtained from two different urban apiaries in Cracow (UEP1 and UEP3) showed a large diversity in the content of bioactive components, which may be due to the variability of vegetation around the apiaries.

### 2.2. Antioxidant Activity of Urban Propolis Extracts

Antioxidant activity was tested in two ways with the use of ABTS and DPPH radicals, as well as by the FRAP technique. The obtained results are presented in Table 2.

The results show a similar antioxidant activity of the urban propolis extracts. ABTS radical scavenging ranged from 16.80 to 51.53 mg Te/mL, DPPH radical scavenging ranged from 7.54 to 22.13 mg Te/mL. The reduction of FRAP occurred in the range of 10.93–29.55 mg Te/mL. The results of the research confirmed the strong antiradical potential of urban propolis by the DPPH and ABTS tests, as well as the reducing capacity expressed by the FRAP method.

**Figure 3:**
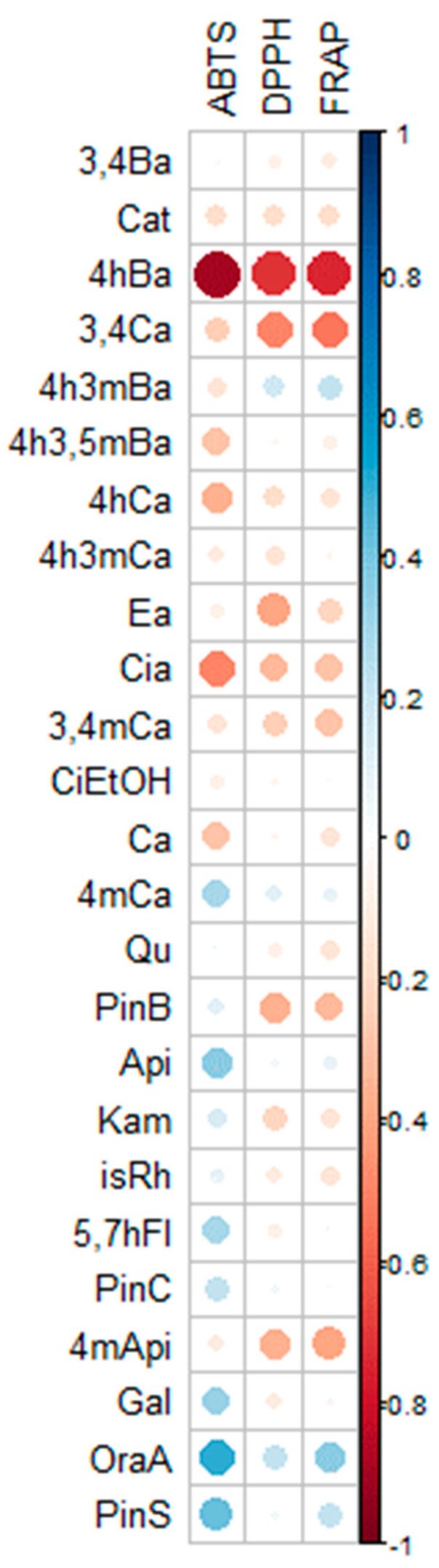
Spearman rank correlation plot based on antioxidants activity and chemical composition of urban propolis extracts.

Significant negative correlations (Figure 3) were observed between the content of 4-hydroxybenzoic acid (4hBa) and cichoric acid (Cia) and the value of antioxidant activity determined by all methods. Among the bioactive components of urban propolis, 3,4-dihydroxycinnamic acid (3,4Ca), ellagic acid (Ea), pinobanksin (PinB) and methyl-4’-apigenin (4mApi) had the greatest influence on the antioxidant activity determined by the DPPH and FRAP methods.

## 3. Discussion

In recent years, there has been a decrease in the number of honey bees (*Apis mellifera*), e.g., due to the frequent use of pesticides, numerous pathogens and diseases such as *Varroa destructor* mites, growing urban agglomerations, a decline in the number of wild flora and climate change [13]. There is also an increase in interest in urban beekeeping, especially in agglomerations, however, despite the rapid development of urban beekeeping, there is scarce information about bee products that are found in cities [14].

The products of apiaries include various types of honey, waxes, propolis, bee pollen, bee bread, royal jelly [15]. Products produced in urban apiaries are identical to those obtained from typical apiaries outside cities and are safe to eat [14,16]. Currently, honey is obtained in the largest quantities from urban apiaries, which is a better-tested product than the others. Few studies of urban propolis have been carried out so far, and they concerned only the content of harmful substances, e.g., heavy metal content [12].

The conducted research is pioneering in the study of the polyphenolic profile and antioxidant activity of urban propolis. These propolis, due to the location of apiaries in Poland, may have a similar chemical composition and biological activity to propolis from Central and Eastern Europe. European propolis is made mainly from poplar and birch, and these are trees that are also often found in Polish cities and available to bees. The original markers that distinguish poplar propolis from others are: pinostrobin, pinocembrine, pinobanksin and caffeic acid. The specific smell of poplar propolis is caused by volatile compounds of mono and sesquiterpenes, which include: cannidol, β-eudesmol, limonene, eucalyptol and β-pinene [1]. The analysis of various propolis samples showed that its chemical composition is very difficult to standardize. It mainly depends on various phytogeographic features, for example: season, vegetation and environmental conditions [17,18]. So far, more than 420 different chemical substances have been identified in propolis that confer pharmacological properties, including amino acids, aromatic acids, essential oils, polyphenolic compounds and waxes [19,20]. Our previous studies of propolis from agricultural lands showed a similar phenolic profile of the extracts. Similarly, the dominant content of caffeic acid and its derivatives was found. Moreover, cichoric acid was identified in all the tested urban propolis extracts, which was first identified in propolis from agricultural areas in Poland [21,22,23,24]. Research by Wozniak et al. [25] showed a high content of epicatechin, catechin, pinobanksin, myricetin and vanilla and syringic acids in propolis from agricultural areas in Poland, these chemical components were also identified in propolis from urban apiaries. Research by Miłek et al. [26] showed a lower content of bioactive ingredients in propolis extracts from Poland, the content of phenolic acids was at the level of 46–77 µg/g of extract, while the content of flavonoids was at the level of 28–81 µg/g of extract. Chromatographic identification of the ingredients showed the presence of the same active compounds as in propolis from urban apiaries. In Turkish propolis, which is also of the poplar type, the presence of the same chemical components as in Polish urban propolis, i.e., pinocembrin, chrysin, pinobanksin, and galangin, as the dominant flavonoids, as well as, ferulic acid, p-coumaric acid, and trans-cinnamic acid as the dominant phenolic acids [27], which confirms that propolis from urban apiaries does not differ significantly from propolis from apiaries located in agricultural areas. As a result of aerobic cellular respiration, by-products are generated in the form of free radicals. In defense against the harmful effects of the presence of radicals, living organisms use exogenous and endogenous antioxidants. These compounds play a very important metabolic role [28]. Ethanol extracts from propolis are characterized by strong antioxidant properties due to the high content of polyphenols and flavonoids. Polyphenols neutralize the action of free radicals (atoms or molecules with one or more unpaired electrons), which cause very unfavorable changes in the human body. Free radicals appear as a result of ionizing radiation, stress, and infections and may result in later serious diseases such as atherosclerosis, arthritis, cancer, or Alzheimer’s and Parkinson’s diseases. Antioxidants contained in propolis may protect against these diseases [29,30]. It has been shown that the high content of some flavonoids, such as quercetin, kaempferol, and galangin, affects the high antioxidant activity of propolis extracts [31,32]. The antioxidant capacity of flavonoids is mainly related to their chemical structure and, as with phenolic acids, is based on the O-H bond dissociation energy value [33].

Research results by Hafshejani et al. [32] indicate that there is a strong correlation between the content of polyphenols and flavonoids in Iranian propolis and the antioxidant activity tested in the DPPH test, which was also shown for some active ingredients in this study [32]. In turn, Tumbarski et al. [34] showed a stronger correlation between the flavonoid content and the antioxidant activity of Bulgarian propolis in the DPPH test than the total polyphenol content. There was no significant correlation between the content of the same ingredients and the antioxidant activity tested using the FRAP technique. Some authors conclude that the FRAP method has limitations and needs to be modified. The fact that it is based on an aqueous solution (acetate buffer) limits the method to hydrophilic substances, while some components of propolis with antioxidant properties (e.g., terpenes) are hydrophobic [33].

## 4. Materials and Methods

### 4.1. Urban Propolis Sample

**Figure 4:**
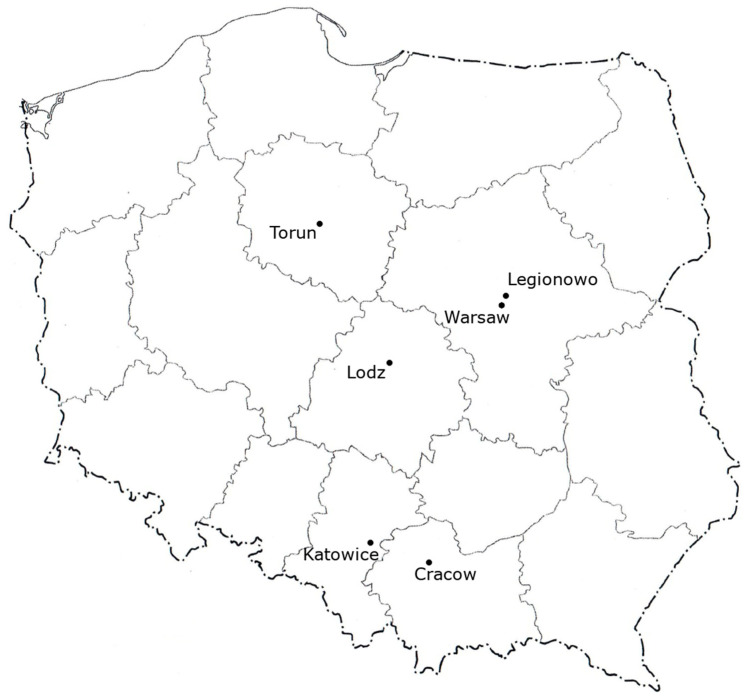
Spearman rank correlation plot based on antioxidant activity and chemical composition of urban propolis extracts.

Propolis samples were obtained from six urban apiary locations in Poland in 2019, as indicated in Figure 4. These samples originated from Polish cities; Cracow (50°03′ N, 19°56′ E) and Katowice (50°15′ N, 19°01′ E), which are located in the south of Poland; Warsaw (52°13′ N, 21°00′ E), Legionowo (52°24′ N, 20°55′ E), and Lodz (51°45′ N, 19°28′ E), which are located in central Poland; and Torun (53°00′ N, 18°35′ E) from central northern Poland. Warsaw is the largest urban agglomeration in Poland with an area of 517 km^2^. In terms of area and population, Cracow and Lodz (approximately 300 km^2^) and Katowice and Torun (approximately 120–165 km^2^) are similar cities. Legionowo is a small town with an area of 13 km^2^. All the hives were located on the roofs of buildings with 1–4 floors in city centers; the established apiaries had more than 5 hives. In Cracow, propolis samples were collected from two different locations. Propolis in Legionowo and Torun were collected on gratings used for this purpose, while in other apiaries, it was scraped off the frames and other parts of the hive. Samples were deposited at the Department of Food Biotechnology and Microbiology at the Institute of Food Sciences of the Warsaw University of Life Sciences-SGGW in Poland. Raw samples of propolis were frozen (−20 °C) and mechanically ground.

### 4.2. Preparation of Urban Propolis Extracts

Samples of crude propolis were extracted with a 10-fold volume of 70% ethanol solution. Samples were shaken (200 rpm) at 28 °C for 1 day (Innova 44R, Eppendorf, Hamburg, Germany). Subsequently, the samples were subjected to ultrasound and were treated with an Omni Ruptor 4000 sonicator provided by a titanium microtip with a 3.8 mm diameter (OMNI International, The Homogenizer Company, Kennesaw, GA, USA). The sonication process was performed for 20 min at a power of 210 W and a frequency of 20 kHz in ice and water baths. The obtained dry extracts were filtered using gravity filtration on a Whatman No. 4 filter and then condensed under reduced pressure at 40 °C (Rotavapor R-215, Büchi, Flawil, Switzerland). Samples of urban propolis extracts (UEP 1–UEP 7) were stored at 4 °C.

### 4.3. Phenolic Compounds Determined by HPLC-DAD

#### 4.3.1. Reagents and Standards

Four reference standards were purchased from Sigma Life Science (Merck, Darmstadt, Germany) and ChromaDex^®^ (Irvine, CA, USA). Phosphoric acid 85%, methanol for HPLC ≥ 99.9%, and acetonitrile for HPLC ≥ 99.9% were purchased from Merck, Darmstadt, Germany. Deionized water was produced by WCA R03 DP ECO (Cobrabid Aqua, Warsaw, Poland).

#### 4.3.2. Chromatography Conditions

The Shimadzu Prominence HPLC system was equipped with two LC-20AD pumps, a SIL-20AC HT auto-sampler, a column oven CTO-10AS VP, and a diode-array UV/VIS detector SPD-M20A. The output signal of the detector was recorded using LC solution 1.21 SP1 chromatography software (Shimadzu, Kyoto, Japan). The separation was executed on a C18 reversed-phase column, 100 mm × 4.60 mm, 2.6 μm particles with a solid core and porous outer layer (Kinetex™, Phenomenex^®^, Torrance, CA, USA). The mobile phase was composed of deionized water adjusted to pH 2 with phosphoric acid and filtered with a 0.20 μm nylon membrane filter (Phenex™, Phenomenex^®^, Torrance, CA, USA) and MeCN with the gradient elution system at a flow rate of 2.0 mL × min^−1^. The injection volume was 1 μL. The detection UV wavelength was set at 230, 240, 254, and 280 nm. The column temperature was set at 45 °C. The gradient was used as follows: 0 min—12.5% B; 25.0 min—40% B; 34.0 min—60% B; 37.0 min—95% B; 37.1 min—12.5% B; 40 min—stop. Compounds were identified by retention time as well UV-spectra (190–450 nm) comparison with standards. The content of the determined compounds was calculated in mg per 100 g of dry matter. 

#### 4.3.3. Preparation of Standard Solutions

The standard stock solutions were prepared by separately dissolving with MeOH in a 25 mL volumetric flask according to the ChromaDex’s Tech Tip 0003: Ref-erence Standard Recovery and Dilution and used as standard stock solutions. Working solutions in several concentrations were mixed and applied to calibration curves. The working solutions and undiluted stock solutions were injected (1 μL) on a column in six replicates (*n* = 6) using an auto-sampler.

### 4.4. Antioxidant Analysis of Urban Propolis Extract

To assess the antioxidant properties of samples, spectrophotometric methods were used, which consisted of determining the 2,2-diphenyl-1-picrylhydrazyl radical (DPPH) and the cation radical 2,2-azinobis (3-ethylbenzothiazoline-6-sulfonate) (ABTS); the FRAP assay relied on the reduction of Fe3+–TPTZ (2,4,6-tri (2-pyridyl)-1,3,5-triazine) to produce Fe2+–TPTZ [35,36]. 

#### 4.4.1. Determination of the Antioxidant Capacity against DPPH and ABTS Radicals

Reactions were performed in 96-well plates. Extract (10 µL) and radical solution (250 µL) were added to each well, mixed, and measured for the absorbance of DPPH after 30 min at 515 nm and for ABTS after 6 min at 734 nm (microplate readers from Multiskan Sky, Thermo Fisher Scientific, Waltham, MA, USA) versus 70% ethanol. The antiradical activity was determined from a decrease in the absorbance of the radical solution in the presence of an antioxidant and expressed as mg Trolox/mL of extract. The determination was carried out in triplicate for each extract. 

#### 4.4.2. Determination of the Antioxidant Capacity of FRAP Assay

Reactions were performed in 96-well plates. First, a 180 µL FRAP working solution and 5 µL extract were added, shook well, and incubated at 37 °C for 15 min in the dark. The absorbance was measured at a wavelength of 593 nm (microplate readers from Multiskan Sky, Thermo Fisher Scientific, Waltham, MA, USA) versus 70% ethanol. The FRAP activity was determined from a decrease in the absorbance of the radical solution in the presence of an antioxidant and expressed as mg Trolox/mL of extract. The determination was carried out in triplicate for each extract.

### 4.5. Statistical Analysis

The data were expressed as the mean ± SD (standard deviation). The obtained results were statistically analyzed in the Statistica 13.3 (TIBCO Software Inc., Tulsa, OK, USA). The significance of differences between the mean values was verified with Tukey’s test (*p* < 0.05) and one-way ANOVA. Heatmaps, Pearson’s rank correlation analysis (*p* < 0.05) results, and plots were obtained using the R platform.

## 5. Conclusions

Establishing apiaries in cities is a new trend that is developing very quickly. In order for products from urban apiaries to be consumed, research on their chemical composition and biological activity is required. In this study, it was shown that urban propolis has a high content of bioactive components, including a favorable phenolic profile, which determines their antioxidant activity. Further research on the biological activity, i.e., antimicrobial properties of urban propolis, is necessary, which is important in their future application in the production and preservation of food.

## Figures and Tables

**Table 1:** Chemical composition of urban propolis extracts determined using HPLC-DAD [µg/100 mg dry matter].

Lp.	Compound		UEP 1	UEP 2	UEP 3	UEP 4	UEP 5	UEP 6	UEP 7
1	3,4-Dihydroxybenzoic acid	3,4Ba	18.82 ± 0.04	0.00 ± 0.00	4.11 ± 0.01	8.46 ± 0.00	5.13 ± 0.01	5.68 ± 0.01	7.61 ± 0.05
2	(+)-Catechin	Cat	0.54 ± 0.04	0.09 ± 0.01	0.19 ± 0.00	0.00 ± 0.00	0.00 ± 0.00	0.00 ± 0.00	0.00 ± 0.00
3	4-Hydroxybenzoic acid	4hBa	589.99 ± 12.31	202.33 ± 4.38	66.55 ± 0.83	100.83 ± 2.60	241.93 ± 2.02	166.47 ± 2.58	68.05 ± 2.17
4	3,4-Dihydroxycinnamic acid	3,4Ca	2039.50 ± 65.42	875.55 ± 4.54	474.50 ± 6.59	1035.53 ± 32.95	539.75 ± 23.29	441.47 ± 12.42	968.90 ± 38.20
5	4-Hydroxy-3-Methoxybenzoic acid	4h3mBa	99.89 ± 1.54	117.51 ± 0.39	53.98 ± 1.18	18.89 ± 0.22	52.88 ± 1.96	122.75 ± 2.92	19.45 ± 0.29
6	4-Hydroxy-3,5-Dimethoxybenzoic acid	4h3,5mBa	1007.43 ± 18.44	361.69 ± 7.37	177.18 ± 3.80	234.85 ± 5.02	181.48 ± 2.25	403.11 ± 11.04	213.59 ± 3.82
7	4-Hydroxycinnamic acid	4hCa	5643.57 ± 172.92	4218.83 ± 45.63	2494.79 ± 76.09	989.34 ± 24.55	2334.70 ± 35.99	3212.36 ± 93.14	1815.47 ± 43.87
8	4-Hydroxy-3-Methoxycinnamic acid	4h3mCa	5348.30 ± 145.74	4 152.01 ± 60.07	265.15 ± 8.67	142.55 ± 4.09	233.67 ± 1.00	2 071.96 ± 27.08	986.70 ± 40.01
9	Ellagic acid dihydrate	Ea	176.79 ± 2.13	12.81 ± 0.09	1.43 ± 0.00	1.42 ± 0.03	1.87 ± 0.02	9.04 ± 0.11	56.22 ± 1.71
10	Cichoric acid	Cia	84.29 ± 1.73	56.50 ± 0.62	17.74 ± 0.25	19.87 ± 0.32	37.56 ± 1.60	64.81 ± 2.33	21.68 ± 0.34
11	3,4-dimetoxycinnamic acid	3,4mCa	269.14 ± 7.30	160.58 ± 0.75	19.65 ± 0.12	255.73 ± 7.89	23.68 ± 0.17	54.76 ± 1.66	251.79 ± 5.23
12	Cinnamyl alcohol	CiEtOH	20.42 ± 0.51	11.79 ± 0.28	4.26 ± 0.06	4.49 ± 0.31	3.15 ± 0.08	5.94 ± 0.10	5.12 ± 0.21
13	Cinnamic acid	Ca	312.63 ± 7.40	96.98 ± 2.04	20.26 ± 0.23	110.39 ± 1.31	26.94 ± 0.52	105.93 ± 2.94	40.98 ± 1.34
14	4-Methoxycinnamic acid	4mCa	50.05 ± 1.47	25.77 ± 0.82	5.95 ± 0.11	79.10 ± 2.41	2.27 ± 0.10	7.45 ± 0.17	62.04 ± 2.68
15	Quercetin	Qu	77.15 ± 2.00	118.29 ± 3.74	47.82 ± 1.42	215.41 ± 6.95	58.18 ± 2.59	60.36 ± 2.41	104.25 ± 3.41
16	Pinobanksin	PinB	360.35 ± 11.88	581.94 ± 1.55	288.15 ± 4.03	687.32 ± 81.39	463.26 ± 21.47	268.38 ± 5.21	825.30 ± 40.81
17	Apigenin	Api	140.82 ± 4.22	413.96 ± 7.49	203.29 ± 3.80	302.48 ± 7.87	197.56 ± 1.41	175.60 ± 4.41	366.40 ± 5.79
18	Kaempferol	Kam	110.49 ± 6.89	284.08 ± 3.25	32.41 ± 1.48	241.40 ± 7.83	180.45 ± 1.33	151.64 ± 3.46	285.76 ± 3.91
19	Isorhamnetin	isRh	11.11 ± 0.23	22.28 ± 0.47	10.71 ± 0.10	41.73 ± 3.30	12.36 ± 0.18	11.48 ± 0.29	29.92 ± 1.52
20	5,7-Dihydroxyflavone	5,7hFl	430.33 ± 14.15	1857.32 ± 71.14	575.93 ± 12.23	1338.46 ± 75.94	703.37 ± 17.99	615.75 ± 20.03	2127.38 ± 63.68
21	Pinocembrin	PinC	286.15 ± 6.75	1524.08 ± 56.92	630.58 ± 21.43	2026.67 ± 52.88	1441.33 ± 36.19	746.47 ± 28.76	2022.86 ± 44.32
22	Methyl-4’-Apigenin	4mApi	389.65 ± 20.13	409.34 ± 10.23	292.31 ± 10.07	1771.91 ± 46.71	1217.90 ± 15.34	349.04 ± 4.94	1325.88 ± 38.07
23	Galangin	Gal	160.83 ± 5.26	983.10 ± 43.44	475.33 ± 14.29	933.42 ± 68.34	637.95 ± 26.25	409.61 ± 13.80	1235.31 ± 37.98
24	O-methylated flavone	OraA	41.84 ± 1.07	281.04 ± 7.46	195.28 ± 1.50	46.93 ± 1.20	87.35 ± 2.07	131.82 ± 3.39	242.83 ± 4.44
25	(+/−)-Pinostrobin	PinS	24.45 ± 0.78	442.96 ± 5.31	690.19 ± 12.55	270.78 ± 9.41	727.94 ± 9.90	636.73 ± 28.27	746.98 ± 35.41
	Alcohol		20.42	11.79	4.26	4.49	3.15	5.94	5.12
	Flavan-3-ols		0.54	0.09	0.19	0.00	0.00	0.00	0.00
	Flavanone		24.45	442.96	690.19	270.78	727.94	636.73	746.98
	Flavanonols		646.50	2106.02	918.72	2713.99	1904.59	1014.84	2848.16
	Flavones		1002.65	2961.65	1266.81	3459.78	2206.18	1272.20	4062.48
	Flavonols		359.58	1407.75	566.26	1431.96	888.94	633.10	1655.24
	Total Phenolic acids		15,640.41	10,280.55	3601.29	2996.95	3681.86	6665.79	4512.47
	Total Flavonoids		2033.72	6918.47	3442.18	7876.50	5727.65	3556.87	9312.87
	Total Phenolics		17,674.13	17,199.02	7043.47	10,873.45	9409.51	10,222.66	13,825.34

UEP 1—extract of urban propolis from Cracow, UEP2—extract of urban propolis from Katowice, UEP 3—extract of urban propolis from Cracow (2), UEP 4—extract of urban propolis from Warsaw, UEP 5—extract of urban propolis from Legionowo, UEP 6—extract of urban propolis from Lodz, UEP 7—extract of urban propolis from Torun.

**Table 2:** The results of antioxidant activity analyses in vitro.

	UEP 1 *	UEP 2	UEP 3	UEP 4	UEP 5	UEP 6	UEP 7
	[mg Te/mL]						
ABTS	23.21 ± 1.30 b	26.64 ± 0.58 bc	47.91 ± 0.93 d	33.47 ± 1.94 c	16.80 ± 0.74 a	44.23 ± 0.42 d	51.53 ± 2.29 e
DPPH	7.82 ± 0.45 a	11.71 ± 0.49 b	19.93 ± 0.49 d	19.18 ± 0.45 d	7.54 ± 0.44 a	22.13 ± 0.45 e	17.19 ± 1.23 c
FRAP	13.30 ± 0.22 b	15.97 ± 0.28 c	28.82 ± 0.60 e	23.49 ± 0.19 d	10.93 ± 0.46 a	29.55 ± 0.38 ef	27.41 ± 0.86 de

* UEP 1—extract of urban propolis from Cracow, UEP2—extract of urban propolis from Katowice, UEP 3—extract of urban propolis from Cracow, UEP 4—extract of urban propolis from Warsaw, UEP 5—extract of urban propolis from Legionowo, UEP 6—extract of urban propolis from Lodz, UEP 7—extract of urban propolis from Torun. The different letters (a–f) in the row indicate statistically significant differences at *p* < 0.05.

## Data Availability

The data presented in this study are available on request from the corresponding author.

## Sample Availability

Samples of the compounds are not available from the authors.

## References

[1] Wagh V.D. (2013). Propolis: A Wonder Bees Product and Its Pharmacological Potentials. Adv. Pharmacol. Sci..

[2] Ristivojević P., Trifković J., Andrić F., Milojković-Opsenica D. (2015). Poplar-type propolis: Chemical composition, botanical origin and biological activity. Nat. Prod. Commun..

[3] Machado C.S., Mokochinski J.B., de Lira T.O., Oliveira F.d.C.E.d., Cardoso M.V., Ferreira R.G., Sawaya A.C.H.F., Ferreira A.G., Pessoa C., Cuesta-Rubio O. (2016). Comparative Study of Chemical Composition and Biological Activity of Yellow, Green, Brown, and Red Brazilian Propolis. Evid. Based Complement. Altern. Med..

[4] Sforcin J.M., Bankova V. (2011). Propolis: Is there a potential for the development of new drugs?. J. Ethnopharmacol..

[5] Shahinozzaman M., Obanda D.N., Tawata S. (2021). Chemical composition and pharmacological properties of Macaranga-type Pacific propolis: A review. Phytother. Res..

[6] Moore L.J., Kosut M. (2013). Buzz: Urban Beekeeping and the Power of the Bee.

[7] Lorenz S., Stark K. (2015). Saving the honeybees in Berlin? A case study of the urban beekeeping boom. Environ. Sociol..

[8] Ropars L., Dajoz I., Fontaine C., Muratet A., Geslin B. (2019). Wild pollinator activity negatively related to honey bee colony densities in urban context. PLoS ONE.

[9] Roest P.B. (2019). *Bees in the D*: A Message of Conservation from an Urban Environment. Challenges.

[10] Stevenson P.C., Bidartondo M.I., Blackhall-Miles R., Cavagnaro T.R., Cooper A., Geslin B., Koch H., Lee M.A., Moat J., O’hanlon R. (2020). The state of the world’s urban ecosystems: What can we learn from trees, fungi, and bees?. Plants People Planet.

[11] Smith T.J., Saunders M.E. (2016). Honey bees: The queens of mass media, despite minority rule among insect pollinators. Insect Conserv. Divers..

[12] Alqarni A.S., Rushdi A.I., Owayss A.A., Raweh H.S., El-Mubarak A.H., Simoneit B.R.T. (2015). Organic Tracers from Asphalt in Propolis Produced by Urban Honey Bees, Apis mellifera Linn. PLoS ONE.

[13] Cox-Foster D.L., Conlan S., Holmes E.C., Palacios G., Evans J.D., Moran N.A., Quan P.-L., Briese T., Hornig M., Geiser D.M. (2007). A Metagenomic Survey of Microbes in Honey Bee Colony Collapse Disorder. Science.

[14] Nicewicz Ł., Nicewicz A.W., Kafel A., Nakonieczny M. (2021). Set of stress biomarkers as a practical tool in the assessment of multistress effect using honeybees from urban and rural areas as a model organism: A pilot study. Environ. Sci. Pollut. Res..

[15] Adaškevičiūtė V., Kaškonienė V., Kaškonas P., Barčauskaitė K., Maruška A. (2019). Comparison of Physicochemical Properties of Bee Pollen with Other Bee Products. Biomolecules.

[16] Nicewicz A.W., Nicewicz Ł., Pawłowska P. (2021). Antioxidant capacity of honey from the urban apiary: A comparison with honey from the rural apiary. Sci. Rep..

[17] Bankova V. (2005). Chemical diversity of propolis and the problem of standardization. J. Ethnopharmacol..

[18] Puspha T.C., Reddy M.S. (2019). Pharmacological potentials of Propolis in urban landscapes. GSC Biol. Pharm. Sci..

[19] Hodel K.V.S., Machado B.A.S., Santos N.R., Costa R.G., Menezes-Filho J.A., Umsza-Guez M.A. (2020). Metal Content of Nutritional and Toxic Value in Different Types of Brazilian Propolis. Sci. World J..

[20] Yong H., Liu J. (2021). Active packaging films and edible coatings based on polyphenol-rich propolis extract: A review. Compr. Rev. Food Sci. Food Saf..

[21] Pobiega K., Kraśniewska K., Przybył J.L., Bączek K., Żubernik J., Witrowa-Rajchert D., Gniewosz M. (2019). Growth Biocontrol of Foodborne Pathogens and Spoilage Microorganisms of Food by Polish Propolis Extracts. Molecules.

[22] Pobiega K., Przybył J.L., Żubernik J., Gniewosz M. (2020). Prolonging the Shelf Life of Cherry Tomatoes by Pullulan Coating with Ethanol Extract of Propolis During Refrigerated Storage. Food Bioprocess Technol..

[23] Pobiega K., Igielska M., Włodarczyk P., Gniewosz M. (2021). The use of pullulan coatings with propolis extract to extend the shelf life of blueberry (*Vaccinium corymbosum*) fruit. Int. J. Food Sci. Technol..

[24] Gniewosz M., Pobiega K., Kraśniewska K., Synowiec A., Chaberek M., Galus S. (2022). Characterization and Antifungal Activity of Pullulan Edible Films Enriched with Propolis Extract for Active Packaging. Foods.

[25] Woźniak M., Sip A., Mrówczyńska L., Broniarczyk J., Waśkiewicz A., Ratajczak I. (2023). Biological Activity and Chemical Composition of Propolis from Various Regions of Poland. Molecules.

[26] Miłek M., Ciszkowicz E., Tomczyk M., Sidor E., Zaguła G., Lecka-Szlachta K., Pasternakiewicz A., Dżugan M. (2022). The Study of Chemical Profile and Antioxidant Properties of Poplar-Type Polish Propolis Considering Local Flora Diversity in Relation to Antibacterial and Anticancer Activities in Human Breast Cancer Cells. Molecules.

[27] Guzelmeric E., Sipahi H., Özhan Y., Hamitoğlu M., Helvacıoğlu S., Düz G., Akyıldız I.E., Yaman B.K., Hazar M., Dilsiz S.A. (2023). Comprehensive estrogenic/anti-estrogenic, anticancer, mutagenic/anti-mutagenic, and genotoxic/anti-genotoxic activity studies on chemically characterized black poplar and Eurasian aspen propolis types. J. Pharm. Biomed. Anal..

[28] Wołosiak R., Drużyńska B., Derewiaka D., Piecyk M., Majewska E., Ciecierska M., Worobiej E., Pakosz P. (2022). Verification of the Conditions for Determination of Antioxidant Activity by ABTS and DPPH Assays—A Practical Approach. Molecules.

[29] Siheri W., Alenezi S., Tusiimire J., Watson D.G., Alvarez-Suarez J. (2017). Bee Products—Chemical and Biological Properties. The Chemical and Biological Properties of Propolis.

[30] Ebiloma G.U., Ichoron N., Siheri W., Watson D.G., Igoli J.O., De Koning H.P. (2020). The Strong Anti-Kinetoplastid Properties of Bee Propolis: Composition and Identification of the Active Agents and Their Biochemical Targets. Molecules.

[31] Cao X.-P., Chen Y.-F., Zhang J.-L., You M.-M., Wang K., Hu F.-L. (2017). Mechanisms underlying the wound healing potential of propolis based on its in vitro antioxidant activity. Phytomedicine.

[32] Fathi Hafshejani S., Lotfi S., Rezvannejad E., Mortazavi M., Riahi-Madvar A. (2023). Correlation between total phenolic and flavonoid contents and biological activities of 12 ethanolic extracts of Iranian propolis. Food Sci. Nutr..

[33] Paula V.B., Estevinho L.M., Cardoso S.M., Dias L.G. (2023). Comparative Methods to Evaluate the Antioxidant Capacity of Propolis: An Attempt to Explain the Differences. Molecules.

[34] Tumbarski Y., Todorova M., Topuzova M., Gineva G., Yanakieva V., Ivanov I., Petkova N. (2023). Comparative Study on Physicochemical, Antioxidant and Antimicrobial Properties of Propolis Collected from Different Regions of Bulgaria. J. Apic. Sci..

[35] Wiktor A., Chadzynska M., Rybak K., Dadan M., Witrowa-Rajchert D., Nowacka M. (2022). The Influence of Polyols on the Process Kinetics and Bioactive Substance Content in Osmotic Dehydrated Organic Strawberries. Molecules.

[36] Xiao F., Xu T., Lu B., Liu R. (2020). Guidelines for antioxidant assays for food components. Food Front..

